# Neuroimaging in Visual Snow - A Review of the Literature

**DOI:** 10.3389/fopht.2022.758963

**Published:** 2022-03-11

**Authors:** Subahari Raviskanthan, Jason C. Ray, Peter W. Mortensen, Andrew G. Lee

**Affiliations:** ^1^ Department of Ophthalmology, Blanton Eye Institute, Houston Methodist Hospital, Houston, TX, United States; ^2^ Department of Neurology, Alfred Health, Melbourne, VIC, Australia; ^3^ Department of Neuro-Ophthalmology, Royal Victorian Eye and Ear Hospital, Melbourne, VIC, Australia; ^4^ Department of Neuroscience, Monash University, Clayton, VIC, Australia; ^5^ Department of Neurology, Austin Health, Heidelberg, VIC, Australia; ^6^ Department of Ophthalmology, Weill Cornell Medicine, New York, NY, United States; ^7^ Department of Neurology, Weill Cornell Medicine, New York, NY, United States; ^8^ Department of Neurosurgery, Weill Cornell Medicine, New York, NY, United States; ^9^ Department of Ophthalmology, University of Texas Medical Branch, Galveston, TX, United States; ^10^ Department of Ophthalmology, University of Texas MD Anderson Cancer Center, Houston, TX, United States; ^11^ Department of Ophthalmology, Texas A and M College of Medicine, Bryan, TX, United States; ^12^ Department of Ophthalmology, The University of Iowa Hospitals and Clinics, Iowa City, IA, United States

**Keywords:** visual snow, neuroimaging, lingual gyrus, occipital cortex, visual cortex

## Abstract

Since the first description of visual snow syndrome (VSS) in 1995, there has been increasing interest particularly within the past 5-10 years in phenotyping the condition and differentiating it from conditions such as migraine with aura and hallucinogen persisting perception disorder. Structural and functional neuroimaging has provided valuable insights in this regard, yielding functional networks and anatomical regions of interest, of which the right lingual gyrus is of particular note. Various modalities, including functional magnetic resonance imaging (fMRI), positron emission tomography (PET), and single photon emission computed tomography (SPECT), have all been studied in patients with visual snow. In this article, we conduct a comprehensive literature review of neuroimaging in VSS.

## 1 Introduction

The clinical manifestations of visual snow syndrome (VSS) were first described by Liu et al. in 1995, who reported a cohort of ten patients with persistent positive visual phenomena that lasted from two months to five years without evidence of migrainous infarction (1). The symptoms reported included positive visual hallucinations of simple geometric forms, transient loss of vision, as well as more complex visual distortions including palinopsia and associated altered body perceptions (1). Since this first report, there has been increasing interest in the condition phenomenologically and it was first formally described as visual snow in 2014 (2, 3). The International Classification of Headache Disorders, third edition (ICHD-3) classifies VSS as a complication of migraine, however notes that further research is required, and it may not be part of the migraine spectrum per se, but rather a common comorbid disorder with shared characteristics (4). The ICHD-3 diagnostic criteria for VSS requires the presence of dynamic, continuous, tiny dots throughout the entire visual field (visual snow) for greater than three months, with the presence of at least two additional features (palinopsia, enhanced entopic phenomena, photophobia, and/or nyctalopia), and that the presentation is not better accounted for by another condition (2, 4). The underlying pathophysiology of VSS remains unclear, but multiple hypotheses have been proposed involving abnormal processing downstream from the primary visual cortex, and neuronal hyperexcitability (5–8). Increasing research, and employment of advanced neuro-imaging has provided invaluable insights into VSS. This article reviews the literature of neuro-imaging in VSS, providing a summary of abnormal findings (**Table 1**) and discusses possible insights for the condition.

**Table 1:** Summary of neuroimaging studies and their findings performed in patients with visual snow syndrome.

Authors	Year	Number of Subjects	Imaging modality	Findings
**Structural MRI**				
Aldusary et al. (5)	2020	19 VSS patients 16 matched controls	MRI	3 VSS patients with increased left occipital volume 5 VSS patients with left occipital bending 2 VSS patients with right occipital bending Higher gray matter volume in right lingual gyrus in VSS patients compared to controls Correlation between duration of VSS and right lingual gyrus GMV
Yildiz et al. (9)	2019	17 VSS patients 12 matched controls	MRI (14 VSS patients)	4 VSS patients with left occipital bending
Schankin et al. (10)	2020	17 VSS patients 17 matched controls	MRI with voxel based morphometry	Patients with visual snow had increased grey matter volume in the adjacent lingual gyrus-fusiform gyrus junction, right middle temporal gyrus, right parahippocampal gyrus, right anterior cingulate cortex. Patients noted to have both increased and decreased grey matter volume in the left superior temporal gyrus.
Puledda et al. (11)	2020	24 VSS patients 24 matched controls	Volumetric MRI	Increased GMV in left primary visual cortex and left cerebellum
**Functional MRI (BOLD, MRS, MRI perfusion)**				
Puledda et al. (8)	2020	26 VSS patients 26 matched controls	fMRI (BOLD)	BOLD activation involving bilateral primary and secondary visual cortices BOLD inactivation in the left insular region when VSS patients looking at simulated VS
Aldusary et al. (5)	2020	19 VSS patients 16 matched controls	fMRI (BOLD)	Hyperconnectivity between the: Left anterior inferior temporal gyrus (AITG) – left posterior temporal fusiform gyrus Right AITG – right anterior temporal fusiform gyrus Left posterior superior temporal gyrus – right inferior occipito-temporal gyrus Left angular gyrus – left lateral prefrontal cortex Right frontal eye field – right angular gyrus Left inferior frontal gyrus – left middle frontal gyrus
Puledda et al. (12)	2021	24 VSS patients 24 matched controls	fMRI (BOLD)	Greater connectivity between right pulvinar, right post central gyrus and supramarginal gyrus. Reduced connectivity between pulvinar and bilateral caudate nuclei Reduced V5 to posterior cingulate cortex. Decreased connectivity between cerebellum and medial precuneus During tasks: Greater connectivity between pulvinar and right lingual gyrus Greater coupling in right V5 and ipsilateral post central/pre central gyri, SMG, premotor cortex, supplemental motor cortex, frontal eye fields. Greater connectivity in V5 to right cuneus/precuneus, Brodmann areas 17, 18, 19, frontal eye field, SMG, premotor cortex, SMA, superior parietal lobule, intraparietal sulcus
Puledda et al. (8)	2020	25 VSS patients 25 matched controls	MRS	Increased lactate levels and glutamate (glutamate not statistically significant) in the right lingual gyrus
Jager et al. (13)	2005	Case series of 2 patients	MRI Perfusion	No abnormalities on perfusion MRI
Puledda et al. (14)	2021	24 VSS patients 24 matched controls	MRI perfusion (Pseudo continuous arterial spin labelling)	Bilateral clusters of increased CBF in VSS patients in the cuneus, precuneus, inferior parietal lobule, superior parietal lobule, supplementary motor area, frontal eye fields, premotor cortex, posterior cingulate cortex, middle frontal gyrus, angular gyrus, post central gyrus, middle and superior occipital lobules. Left sided increased CBF in the primary auditory cortex, fusiform gyrus, area VI in the cerebellum, and supramarginal gyrus. During tasks, increased activation of the right anterior insula
**SPECT**				
Liu et al. (1)	1995	2 patients with VS symptoms	SPECT	Hypometabolism in the bilateral parietal lobes in one patient, hypometabolism in the bilateral parieto-occipital lobes in the other patient.
Shibata et al. (15)	2020	3 patients with VS symptoms	SPECT	Hypometabolism in the bilateral occipital lobes and fusiform gyri in one patient, hypometabolism in frontal lobes in one patient, one patient normal.
**PET**				
Schankin et al. (10)	2020	17 patients 17 age matched controls	PET	Right lingual gyrus hypermetabolism – Brodmann area 19 Hypometabolism of the right superior temporal gyrus, left inferior parietal lobule Hypermetabolism of the left anterior lobe of the cerebellum

BOLD, blood oxygen level dependent; CBF, cerebral blood flow; fMRI, functional magnetic resonance imaging; MRI, magnetic resonance imaging; MRS, magnetic resonance spectroscopy; PET, positron emission tomography; SMA, supplemental motor area; SMG, supramarginal gyrus; SPECT, single photon emission computed tomography; VS, visual snow; VSS, visual snow syndrome.

## 2 Neuroimaging in VSS

### 2.1 Structural Neuroimaging

While investigation of VSS with cranial computerized tomography has not identified any structural abnormalities, the literature on MRI findings is more heterogeneous (16). The current literature, primarily consisting of case level evidence, includes 85 reports of patients with VSS and no evidence of structural abnormality on cranial MRI (7, 13, 16–23). In cases reported to have a structural abnormality, the most common finding related to the occipital cortex and the cerebellum (5, 9, 11, 24).

#### 2.1.1 Occipital Bending

Changes in the occipital lobe including an asymmetric lateral ventricle can produce MRI findings where one occipital lobe wraps around the other and has been termed “occipital bending”. There are 12 case reports in the literature of occipital bending on cranial MRI (ten left, two right sided), with one case associated with hemispheric asymmetry (5, 9, 24). A case-control study compared brain MRI of 19 patients with VSS to 16 matched controls. Increased left occipital volume was noted in three patients, while occipital bending was seen in five patients on the left, and two on the right (5). Another report of 17 patients with VSS and 12 matched controls found left occipital bending in four subjects (9). The overall prevalence of this finding in VSS patients is unknown due to the relatively small populations involved in these studies, but has not been noted in any of the control patients.

Occipital bending, the phenomenon of the occipital lobe crossing the antero-posterior axis, has also been reported in several psychiatric conditions, including depression, bipolar disorder, and schizophrenia (25–29). Several theories have been postulated for the cause of this observation, including enlargement of a lateral ventricle, greater cerebrospinal fluid volumes, or increased or decreased white matter in other areas, causing secondary asymmetric different pressure in the occipital cortex. Further research is required to elucidate this. Of the twelve patients reported in the literature with VSS and occipital bending, at least four had a comorbid diagnosis of depression (the data was not available in four cases) (5, 9, 24). Further research is required therefore, to elucidate whether this finding is related to VSS or psychiatric comorbidity, as well as the underlying cause of the occipital bending.

#### 2.1.2 Increased Gray Matter Volume

Increased gray matter volume (GMV) in the lingual gyrus has been found on volumetric MRI in 19 patients with VSS compared to 16 controls (5). A statistically significant correlation between right lingual gyral gray matter volume and disease duration was also noted (5). A second study of 17 patients with VSS with matched controls also noted increased GMV in the lingual gyrus-fusiform gyrus junction, which correlated with hypermetabolism on PET (discussed in more detail below), suggesting both a structural and functional association with VSS (10). This study also found increased GMV in the right middle temporal gyrus, right parahippocampal gyrus, and right anterior cingulate cortex, though these did not have abnormal findings on PET. Both increased and decreased GMV was noted in the left superior temporal gyrus in some of these patients (10).

The lingual gyrus, located on the inferomedial aspect of the occipital lobe, has a role in V1 and V2 visual field representation of the contralateral superior quadrant, encoding complex images, processing visual information such as parts of human faces and maintaining images in working memory, which may explain its involvement in VSS (30–32). Increased GMV in the lingual gyrus has been previously reported to be associated with divergent thinking and creativity, possibly due to increased synaptic connections (33). Supporting this hypothesis of reactive GMV change, a 2018 study of 19 healthy subjects underwent an MRI pre and post 45 minutes of sensory stimulation, demonstrated increase in GMV in brain regions receiving afferent input from the stimulated body site (34). GMV in the lingual gyrus specifically, along with other areas of the visual pathway, are increased in airline pilots and athletes, further supporting the hypothesis (35, 36).

In a separate study of 24 patients with VSS compared to matched controls, increased GMV was reported in the left primary visual cortex and left cerebellum (11). The laterality of this finding was speculated to be a statistical, not a morphological observation, as increased GMV was also noted on the contralateral hemisphere on *post-hoc* analysis with altered significance thresholds. PET has also shown hypermetabolism in the visual cortex previously (discussed further below), further supporting the hypothesis of occipital cortex involvement (11). Of note however, migraine is a common comorbidity seen in up to 80% of patients with VSS (36, 37). GMV in migraine has been variably reported to be increased in the lingual, fusiform and parahippocampal gyri, decreased in the V1, V2, V3, and V5 visual areas, or show no different to controls (38–40). Further research is required to clarify these observations, and the significance of GMV between these two conditions.

### 2.2 Functional MRI

#### 2.2.1 Blood Oxygenation Level Dependent Imaging

Functional magnetic resonance imaging (fMRI) measures changes in brain metabolism, with the intention of differentiating metabolic changes related to specific tasks from other unregulated brain processes (41). The use of blood oxygenation level dependent (BOLD) activity to quantify areas of activation during particular tasks is one sequence of fMRI. Puledda et al. investigated 24 patients with VSS and 24 controls with fMRI while looking at a dark screen or simulated visual snow images. Both groups were found to have BOLD activation in the primary and secondary visual cortices when looking at the visual snow images (8). Additionally, the VSS group also showed BOLD deactivation in the left insula when looking at the visual snow images compared to the dark screen and the control group, also noted on the right with decreased significance thresholds (8). Cluster analysis within groups also showed BOLD deactivation with the snow task in the middle frontal gyrus, superior frontal gyrus, frontal eye fields, supramarginal gyrus, frontal operculum, and right insula with clusters from both groups also showing periventricular area BOLD deactivation (8).

Puledda et al. reviewed 24 patients and matched controls with fMRI at 8 seed points – the right pulvinar, right V1, right V5, right lingual gyrus, left cerebellum nodule VI, posterior midcingulate cortex/posterior cingulate cortex; left precuneus and right insula, locations based on findings in previous studies (12). Hyperconnectivity was noted between multiple regions, including the right pulvinar to the right postcentral and supramarginal gyrus, V5 to the right cuneus, precuneus, Brodmann visual areas 17, 18, 19, frontal eye fields, supramarginal gyrus, premotor cortex, supplementary motor area, superior parietal lobule, intraparietal sulcus, left precuneus to the right precentral gyrus and frontal eye fields, posterior cingulate gyrus to the bilateral medial precuneus, and from the cerebellum to the right lateral precuneus, right superior parietal lobule, and post central gyrus. When patients were exposed to the VS simulating task, there was additional hyperconnectivity noted between the right pulvinar and right lingual gyrus. Areas of decreased connectivity between the pulvinar and bilateral caudate nuclei, right V5 to posterior cingulate cortex and the right temporo-parietal junction, and the cerebellum to the posterior cingulate cortex and medial precuneus. *Post hoc* analyses adjusting for migraine showed most of these cluster differences remained statistically significant, except for the cerebellar seed findings.

Another study by Aldusary et al. assessed resting state functional connectivity of the BOLD signal comparing 19 patients with VSS to 16 controls (5). Following adjustment for multiple covariates, the authors found hyperconnectivity in the resting state between multiple brain regions. These included the left anterior inferior temporal gyrus (AITG) to left posterior temporal fusiform gyrus, right AITG to the right anterior temporal fusiform gyrus, left posterior superior temporal gyrus to the right inferior occipital temporal gyrus, left angular gyrus to the left lateral prefrontal cortex, right frontal eye field to the right angular gyrus, and the left inferior frontal gyrus to the left middle frontal gyrus (5). Of note, no hyperconnectivity to the right lingual gyrus was noted.

The findings of the within-group VSS cluster analysis by Puledda showing areas of BOLD deactivation with activity in areas where there was BOLD activation in Aldusary’s cohort of patients at rest (frontal eye field, middle frontal gyrus) might suggest these areas are of more significance.

#### 2.2.2 MRI Perfusion

MRI perfusion has been used in migraine, malignancy, and stroke to identify functional abnormalities not visible on structural CT or MR imaging (42). No abnormalities on contrast enhanced perfusion MRI have been noted in VSS patients, though only one small case series of two patients has been reported (13).

Cerebral blood flow (CBF) maps with three-dimensional pseudo-continuous arterial spin labelling (pASL) have also been used to assess areas of functional activation in patients with VSS. Twenty-four VSS patients, matched with twenty-four age and sex matched controls underwent perfusion imaging with pASL during both a visual task (simulated visual snow image) and a control image (12). At baseline, multiple clusters of increased CBF were seen bilaterally in the VSS patients in the cuneus, precuneus, inferior parietal lobule, superior parietal lobule, supplementary motor area, frontal eye fields, premotor cortex, posterior cingulate cortex, middle frontal gyrus, angular gyrus, post central gyrus, middle and superior occipital lobules. Increased CBF were also noted on the left hemisphere in the primary auditory cortex, fusiform gyrus, area VI in the cerebellum, and supramarginal gyrus. After the visual snow stimulus, the posterior cingulate cortex and the left inferior parietal lobule did not show a significant difference, but the other listed locations did. *Post hoc* analysis in VSS patients without migraines only revealed increased cerebral blood flow in the left superior temporal gyrus, right superior parietal lobule, cuneus, and precuneus, and comparisons to migraine patients previously studied with similar imaging protocols showed right precentral gyrus and right precuneus activation (14). When performing tasks, the VSS patients had increased CBF to the right anterior insula compared to the control group, and that the control group had deactivation of this region (12).

The insula, the most consistently implicated structure in functional MRI, has been shown to have multiple roles in processing of visual stimuli, including initiation of visual motion imagery neurofeedback in patients without VSS (14). Activation of the insular cortex has been shown to be involved in the determination of the orientation of object movement, and noted in speech processing of lip movements without audible sounds (43). Similarly, remodeling of the anterior and posterior insula on fMRI have been noted in patients with blindness, both congenital and acquired (44). Within the VSS populations, as discussed above, findings of BOLD deactivation primarily in the left insula, as well as increased CBF in the right anterior insula have been noted. One hypothesis is that insular inactivation during visual snow like tasks may relate to baseline increased activation of the insula, or reduction in activity with the task; the former explanation is thought to better match with the insula involvement in the salience network (8). It was also noted that external stimulation of visual snow may not have the same neuroimaging correlates as the intrinsic snow perceived by the VSS cohort, and this may also explain the insula findings.

#### 2.2.3 MR Spectroscopy

Puledda et al. also utilized MR spectroscopy (MRS) to evaluate the metabolic profile of patients with VSS. The authors studied 25 patients who had prior fMRI BOLD imaging and 25 matched controls who underwent MRS while looking at simulated visual snow and control images. The study found a significant increase in lactate levels in the right lingual gyrus in the VSS group, and a non-significant increase in glutamate levels (p=0.06) (8). These findings were postulated to be related to inefficiencies in cellular metabolism, resulting in increased anaerobic glycolysis, possibly coupled with increased energy demand from activation of the region, thereby raising lactate levels (8). The uptrend in glutamate, an excitatory neurotransmitter, which trended towards statistical significance, was also thought to represent hyperexcitability of the region. Combined with the BOLD fMRI, this was thought to represent increased activation of the lingual gyrus.

### 2.3 Single Photon Emission Computed Tomography

Hypometabolism on SPECT was reported in two of the patients in the initial series of patients reported by Liu. One patient was reported to have bilateral parietal, and the other bilateral parieto-occipital hypometabolism (1). A second case series of VSS patients reported one patient with bilateral occipital and fusiform gyri hypometabolism, one with mild frontal hypoperfusion, and a third patient with a normal SPECT scan (15). The relative heterogeneity of these findings and lack of age-matched controls limits the interpretation of this data.

### 2.4 Positron Emission Tomography

One case-control study has been published of patients with VSS who have undergone PET. In the study, 17 patients, and 17 matched controls underwent a PET scan. None of the control patients had comorbid migraine, however 82% of patients with visual snow also had migraine (10). The visual snow group was found to have right lingual gyrus hypermetabolism, as well as a trend towards hypermetabolism of the anterior lobe of the left cerebellum, after adjusting for the presence of typical migraine aura within the groups (10).

## 3 Discussion

### 3.1 Current Proposed Models of VSS

Whilst the definite underlying pathophysiology of visual snow is still not completely clear, the studies above support the hypothesis of multiple network involvement, including the visual, attentional, and salience networks. Abnormalities in all 3 of these pathways, both at rest, and during tasks, are consistent with a multi-domain aetiology, however need to be taken into consideration with the limitations below.

### 3.2 Limitations Within the Literature

Whilst some consistent findings have been noted through multiple studies, in particular the involvement of the right lingual gyrus, methodological challenges and differences within these studies need to be acknowledged, and are also summarised in **Table 2**.

**Table 2:** Patient characteristics in visual snow syndrome studies.

Study	Age Criteria	Migraine	Other Comorbidities	HPPD/recreational drug use	Pregnancy
Aldusary et al. (5)	Over 18 years old	Not an exclusion criterion for VSS or control group. 53% of VSS patients had migraine, 31% of healthy controls had migraine.	Comorbid neurodegenerative disorders excluded	Not an exclusion criterion for VSS or control group	All pregnant patients excluded
Yildiz et al. (9)	18 – 60 years old	Patients on migraine prophylaxis were excluded from the control group. Visual snow patients were divided into VSS plus migraine, and VSS without migraine	All patients with ophthalmic, neurological, or psychiatric illnesses were excluded	Patients with a history of drug abuse were excluded	Excluded
Schankin et al. (10)	Patients aged 18 – 50 years old	Patients with migraine > every 2 months, or migraine with aura were excluded from the control group.	Comorbid ophthalmic disorders	Any history of hallucinogen use, or recreational drug use within the prior 6 months	Excluded
Puledda et al. (8, 11, 12, 14)	Patients aged 20 – 60 years old	15 of the 24 VSS patients had migraine. No migraine patients in the control group. A second model was run with migraine as a covariate	No comorbidities or regular medications were allowed in the control group.	Patients who had a history of recreational drug use were excluded	Not discussed

HPPD, Hallucinogen persistent perception disorder; VSS, visual snow syndrome.

#### 3.2.1 Migraine as a Comorbidity

Whilst the prospective matched neuroimaging studies utilize age and sex matched controls, the approach to the migraine comorbidity is more variable. The prevalence of migraine in visual snow patients is reported as high as 80% in some studied populations, and whether this could be a potential confounder is still unclear. Studies by Puledda et al, which contribute a substantial proportion of the neuroimaging literature in VSS patients, excluded migraineurs from the control group, although some of the studies utilised a second model with migraine as a covariate (8, 11, 12, 14). Schankin et al. also excluded migraineurs/frequent migraineurs from the control group, whereas Aldusary et al. had migraineurs in both the VSS and control groups, and Yildiz et al. had a VSS group with migraine and one without migraine (5, 9, 10). Although there were no statistically significant differences in clinical characteristics in the VSS with and without migraine groups in the Yildiz study, larger cohorts of VSS patients have noticed different clinical characteristics within the migraineurs and non migraineurs, strengthening the argument for this being a potential confounder (9, 45).

#### 3.2.2 Relationship to Hallucinogen Persistent Perception Disorder/Recreational Drug Use

Hallucinogen persistent perceptual disorder (HPPD) and recreational drug use, can both produce symptoms similar to VSS. By the ICHD-3 diagnostic criteria for VSS, if HPPD is suspected, a diagnosis of VSS cannot be made. Most studies excluded all patients with a history of recreational drug use, to minimize HPPD patients from being included in the patient cohort, however Aldusary et al. did not have this as an exclusion criteria, again highlighting the heterogeneity of the populations within the studies (5).

#### 3.2.3 Age/Comorbidities

There were slight variations in inclusion criteria for age, but most studies including patients 18 – 20 years to 50 – 60 years in age. Similarly, minor variations in acceptable comorbidities existed between studies, but generally patients with major comorbidities, especially with neurological, ophthalmic, or psychiatric manifestations were excluded. Pregnant patients were excluded from all groups except in studies by Puledda et al, where pregnancy status was not specifically discussed, although patients who could not have MRI were excluded from these trials, suggesting pregnant patients may have been excluded.

## 4 Conclusion

Our current understanding of VSS remains ill-defined but multiple potential mechanisms and localizations have been proposed. Some of these hypotheses appear to have clinico-radiologic correlation on functional and structural neuro-imaging studies including fMRI, PET, and SPECT. To date, the current literature describes mainly heterogeneous imaging findings based upon case reports, small case series, and a few case-control studies. The most consistently reported neuroimaging findings in VSS involve the lingual gyrus and occipital cortex and supports the hypothesis that VSS is a downstream visual processing disorder. We recognize that the lingual gyrus contains the V1 and V2 visual field representation of the contralateral superior quadrant for most of its extent but also encodes complex images, processes specific visual information including human faces, and maintains images in working memory. Although the majority of anatomical findings described in the literature reference the entire lingual gyrus without reference to the exact subregion of the gyrus, we believe that future study is necessary to provide putative structure and function correlation in VSS.

Increased GMV, which is observed both in VSS and in normal populations, may be either a marker, or consequence of, the condition. Studies thus far are also somewhat heterogeneous in the patient population, allowing for possible other confounders, especially migraine.

Further research is now required to undertake larger, controlled studies to delineate these early observations, while controlling for confounding conditions such as migraine or psychiatric comorbidity. Longitudinal studies are required to determine if GMV can be correlated with clinical phenotype, duration or severity of symptoms, or response to treatment. Future neuroimaging study of VSS including fMRI, PET, and SPECT will be necessary to define the disorder better and to assess the potential efficacy of any new treatments for VSS.

## Author Contributions

Category 1: Conception and design: SR, JR, PM, and AL. Acquisition of data: SR, JR, PM, and AL. Analysis and interpretation of data: SR, JR, PM, and AL. Category 2: Drafting the manuscript: SR, JR, PM, and AL. Revising it for intellectual content: SR, JR, PM, and AL. Category 3: Final approval of the completed manuscript: SR, JR, PM, and AL. All authors contributed to the article and approved the submitted version.

## Conflict of Interest

The authors declare that the research was conducted in the absence of any commercial or financial relationships that could be construed as a potential conflict of interest.

## Publisher’s Note

All claims expressed in this article are solely those of the authors and do not necessarily represent those of their affiliated organizations, or those of the publisher, the editors and the reviewers. Any product that may be evaluated in this article, or claim that may be made by its manufacturer, is not guaranteed or endorsed by the publisher.
